# Pharmaceutical Applications of Hot-Melt Extrusion: Continuous Manufacturing, Twin-Screw Granulations, and 3D Printing

**DOI:** 10.3390/pharmaceutics11050218

**Published:** 2019-05-07

**Authors:** Mohammed Maniruzzaman

**Affiliations:** 1 School of Life Sciences, University of Sussex, Falmer, Brighton BN1 9QJ, UK; M.Maniruzzaman@austin.utexas.edu or M.Maniruzzaman12@gmail.com; 2 Division of Molecular Pharmaceutics and Drug Delivery, College of Pharmacy, University of Texas at Austin, University Station A1920, Austin, TX 78712, USA

Recently, hot-melt extrusion (HME) techniques have been presented as innovative platforms to produce various pharmaceuticals. HME is an emerging processing technology used primarily for the manufacture of pharmaceutical solid dispersions. It also combines the advantages of a solvent-free process with fewer production steps; HME is both simple to scale up and offers continuous manufacturing applications. A single-unit, HME-based operation, employing heat and mechanical shear, has displayed a significant potential to retain the stability of thermo-labile therapeutics, e.g., proteins. Although HME is as an effective means to manufacture, optimize, and deliver various novel macromolecules and biologics, it has recently been established from a quality by design (QbD) viewpoint as per the recent guidelines issued by the Food and Drug Administration (FDA). Moreover, HME can be coupled with modern manufacturing techniques such as 3D printing to deliver immediate potential for unit dose fabrications. As the guest editor of this special issue of *Pharmaceutics*, it is an honor and privilege to introduce this issue on “Pharmaceutical Applications of Hot-Melt Extrusion”. This theme issue contains 10 original research and 3 critical review papers focusing on various recent and emerging applications of HME techniques, which, I believe, will be of significant interest to readers across both industry and academia.

The paper by Fuenmayor et al. details the complications arising when adopting pharmaceutical grade polymers for fused-filament fabrication in the production of oral tablets by means of fused deposition modelling (FDM) 3D printing. The paper also highlights methods to overcome each issues encountered in fused-filament fabrication with Kollidon® VA64 polymeric carrier [1]. Wesholowski et al. examine a UV/Vis spectrophotometer for its suitability as a process analytical tool (PAT) for the in-line determination of the Residence Time Distribution (RTD) in a HME process. The authors also describe the necessity of using an efficient PAT for continuous processes like HME or twin-screw granulations, according to the framework of Quality-by-Design (QbD), for the inline determination of process parameters or quality attributes of a product [2]. Similarly, Schlindwein et al., report a case study to display the potential of an in-line UV–Vis spectroscopy-based PAT system for early phase product development during pharmaceutical continuous manufacturing following a QbD framework. The authors use a sequential design of experiments (DoE) (screening, optimization, and verification) to gain process understanding for the manufacture of piroxicam (PRX)/Kollidon® VA64 amorphous solid dispersions. The influence of die temperature, screw speed, solid feed rate, and PRX concentration on the critical quality attributes (CQAs) of absorbance and lightness of color (L*) of the extrudates was investigated using multivariate tools [3].

Feng et al. evaluate the combined effect of polymers and surfactants on maintaining the supersaturated state of itraconazole (ITZ) in amorphous solid-dispersion (ASD) systems produced via HME processing. The authors investigate the integration of various surfactants with enteric polymer hydroxypropyl methylcellulose acetate succinate (HPMC AS) to develop polymer–surfactant-based solid dispersion. The study concludes that the amorphous solid dispersion (ASD), which is based on a polymer–surfactant system, could inhibit drug precipitation both in vitro and in vivo [4]. Lauer et al. present an interesting case that utilized a miniaturized extrusion device (MinEx) to manufacture hypromellose acetate succinate type L- (HPMCAS-L) based extrudates containing the model drugs neurokinin-1 (NK1) and cholesterylester transfer protein (CETP). The authors also used plasticizers and assessed their impact on dissolution and solid-state properties. A comparative study with a lab scale extruder revealed that MinEx is a valuable prototyping-screening method and the properties of the extrudates translated to products manufactured in lab-scale extrusion trials for at least eight different formulations [5]. Takabe et al. explore the efficacy of atovaquone against glioblastoma multiforme (GBM) as well as the development of a formulation of atovaquone to improve oral bioavailability, resulting in higher amounts of drug delivered to the brain by means of amorphous solid dispersion of the drug. The authors also assessed a proof-of-concept in vivo exposure study of their optimized formulation and concluded that the enhanced amorphous solid dispersion is promising for providing therapeutically effective brain levels of atovaquone for the treatment of GBM [6].

Salman et al. report two studies on emerging twin-screw granulations. The first study focused on the investigation of the influence of varying barrel fill levels on the mean residence time, granule properties (median size, size distribution, and shape), and tensile strength of tablets. In this study, specific feed load (SFL) (powder feed rate divided by screw speed) and powder feed number (PFN) (i.e., powder mass flow rate divided by the product of screw speed, screw diameter, and the material density in the denominator) were considered surrogates for the barrel fill level. The authors concluded that, at very high fill levels, granule size decreased because of the limited interaction between microcrystalline cellulose (MCC) powder and liquid at high throughput force and short residence time [7]. The same group compared three grades of both lactose and mannitol to determine the granulation mechanism of different grades of two pharmaceutical powders with varying properties (i.e., primary particle size, structure, and compressibility). The authors found that the primary powder morphology played an important role in determining the granule size and structure as well as tablet tensile strength [8]. Bochmann et al. presented a validation for the use of model-based melt viscosity in hot-melt extrusion numerical simulations for the development of amorphous solid dispersion (ASD). For the study, four active pharmaceutical ingredients (APIs) were examined to establish the correlation between Tg and melt viscosity. The authors found that, with few exceptions, the use of model-based melt viscosity in terms of the HME simulation did not reduce the accuracy of the computation outcome [9]. 

In a separate study, Theismann et al. developed an alternative process to spray granulation to prepare high-loaded spherical nicotinamide (NAM) pellets by a wet extrusion and spheronization technique. The authors implemented a QbD approach to model the effect of the process parameters of the extrusion–spheronization process on the roundness, roughness, and useable yield of the obtained pellets. The obtained results were compared with spray granulated NAM particles in terms of their characteristics and their release profile in vitro after the application of an ileocolonic-targeted shellac coating [10]. 

Lowinger et al. thoroughly reviewed the applications of polyurethanes to the development of modified release drug delivery. The authors briefly reported the chemistry of polyurethanes and the mechanisms of drug release from sustained release dosage forms. Additionally, the impact of intrinsic drug properties on release from polyurethane-based formulations, the impact of hydrophilic, water-swelling polyurethanes on drug diffusivity and release rate were highlighted in this critical and timely review paper [11]. Censi et al. reported important physicochemical factors that should be investigated for the design and optimization of a hot-melt extrusion process during different pre-formulation and formulation, and post-formulation phases. The authors highlight the last ten years of research, extending inquiry as broadly as possible on various aspects of HME processing [12]. Lastly, Nokhodchi and co-workers review the working principle of HME and FDM 3D printing, and how these two technologies can be combined for the use of advanced pharmaceutical applications. The authors assert that 3D printing technology has been widely used for rapid prototyping and its interest as a fabrication method has grown significantly across many disciplines. FDM 3D printing technology utilizes filaments manufactured via HME processing. The group concludes that, by introducing HME techniques for 3D printing, filament development can improve the bioavailability and solubility of drugs as well as sustain drug release for a prolonged period [13]. 

The guest editor would like to thank and acknowledge the enormous support and valuable contributions from the authors, without which this authoritative and time special issue would not be possible. 
